# Supplementary material for: Food pricing: A study on the sales of food in Brazilian private schools
Source: PLoS One. 2025 Nov 17;20(11):e0336955. doi: 10.1371/journal.pone.0336955 (PMC12622845; doi:10.1371/journal.pone.0336955)
Supplement: S1 File — This appendix includes additional tables referenced in the manuscript, including the total number of canteens and period of data collection in each capital of Brazilian states and the Federal District (Table S1), the correspondence of the 50 foods and beverages evaluated in the canteens for each item of the list of the Extended Consumer Price Index (IPCA) (Table S2); and identification and approval by the respective ethics committees (Table S3). (DOCX) [file pone.0336955.s001.docx]

**Supplementary material**

**Table S1** – Total number of canteens in private elementary and secondary schools, and period of data collection in each capital of Brazilian states and the Federal District. Food sale in Brazilian Schools (Caeb). 2022-2024.

| **Region of Brazil** | **Cities (Federative Units)** | **N** | **%** | **Data collection period** |
| --- | --- | --- | --- | --- |
| Northeast | Aracaju (Sergipe) | 71 | 3.17 | August 2022 to March 2023 |
|  | Fortaleza (Ceará) | 113 | 5.04 | January 2023 to September 2023 |
|  | João Pessoa (Paraíba) | 65 | 2.90 | August to October 2023 |
|  | Maceió (Alagoas) | 96 | 4.28 | August to November 2023 |
|  | Natal (Rio Grande do Norte) | 75 | 3.35 | August to October 2023 |
|  | Recife (Pernambuco) | 124 | 5.53 | August 2022 to March 2023 |
|  | Salvador (Bahia) | 126 | 5.62 | November 2022 to August 2023 |
|  | São Luís (Maranhão) | 108 | 4.82 | August to December 2023 |
|  | Teresina (Piauí) | 56 | 2.50 | August to October 2023 |
| Southeast | Belo Horizonte (Minas Gerais) | 76 | 3.39 | June to December 2022 |
|  | Rio de Janeiro (Rio de Janeiro) | 200 | 8.92 | June to December 2022 |
|  | São Paulo (São Paulo) | 262 | 11.69 | 10 schools evaluated in November 2023 and the others from February to June 2024 |
|  | Vitória (Espírito Santo) | 15 | 0.67 | August to November 2023 |
| North | Belém (Pará) | 103 | 4.60 | August 2023 to April 2024 |
|  | Boa vista (Roraima) | 16 | 0.71 | March to May 2024 |
|  | Manaus (Amazonas) | 62 | 2.77 | March to June 2024 |
|  | Macapá (Amapá) | 29 | 1.29 | February to April 2024 |
|  | Palmas (Tocantins) | 21 | 0.94 | February to April 2024 |
|  | Porto Velho (Roraima) | 17 | 0.76 | February to April 2024 |
|  | Rio Branco (Acre) | 21 | 0.94 | February to April 2024 |
| Central-West | Federal District | 179 | 7.99 | January to July 2023 |
|  | Campo Grande (Mato Grosso do Sul) | 58 | 2.59 | March to June 2024 |
|  | Cuiabá (Mato Grosso) | 53 | 2.37 | August to November 2023 |
|  | Goiânia (Goiás) | 115 | 5.13 | March to April 2024 |
| South | Curitiba (Paraná) | 81 | 3.61 | February to April 2024 |
|  | Florianópolis (Santa Catarina) | 39 | 1.74 | February to April 2024 |
|  | Porto Alegre (Rio Grande do Sul) | 60 | 2.68 | June to August 2022 |
|  | Total | 2,241 | 100 | - |

**Table S2** – Correspondence of the 50 foods and beverages evaluated in the canteens for each item of the list of the Extended Consumer Price Index (IPCA).

| **Foods and drinks** | **Classification (correspondence according to IBGE's Automatic Recovery System -SIDRA)** |
| --- | --- |
| **UMPCP** | |
| Açaí without sugar or syrup | 1114004.Acaí (emulsion) |
| Coconut water | 1114001.Fruit juice |
| Mineral water (sparkling or still) | 1201007.Soda and mineral water |
| Handmade cookies | 1112003.Cookie |
| Handmade cake | 1112019.Cake |
| Coffee (drip-brewed or espresso) | 1201009.Small coffee |
| Herbal tea (infusion prepared at the canteen) | 1114091.Mate tea (yerba mate) |
| Sweet made from fruits or vegetables | 1104060.Fruit paste |
| Fresh fruit | 1106.Fruits |
| Dried fruit | 1106.Fruits |
| Fruit smoothie with milk | 1111019.Yogurt and yogurt drinks |
| Brazilian cheese puffs | 1112025.Brazilian cheese puffs |
| Sweet or salty popcorn made with fresh kernel | 1201003.Snack |
| Pizza without an ultra-processed filling | 1201003.Snack |
| Simple fruit salad | 1106.Fruits |
| Baked salty snack without an ultra-processed filling | 1201003.Snack |
| Fried salty snack without an ultra-processed filling | 1201003.Snack |
| Sandwich without an ultra-processed filling | 1201003.Snack |
| 100% whole juice - carton, can, or bottle | 1114001.Fruit juice |
| Natural fruit juice (freshly squeezed or processed fruit pulp) | 1114001.Fruit juice |
| Tapioca without an ultra-processed filling | 1201003.Snack |
| Açaí with sugar or syrup | 1114004.Acaí (emulsion) |
| **UpCP** | |
| Açaí with toppings | 1114004.Acaí (emulsion) |
| Cereal bar | 1201003.Snack |
| Soy drink | 1111019.Yogurt and yogurt drinks |
| Yogurt drink and flavored yogurt | 1111019.Yogurt and yogurt drinks |
| Sweet cookie with or without a filling | 1112003.Cookie |
| Ultra-processed cake | 1112019.Cake |
| Bonbon or chocolate bar | 1104023.Chocolate bar and bonbon |
| Breakfast cereal | 1201003.Snack |
| Ready-to-drink tea | 1201007.Soda and mineral water |
| Sweet with ultra-processed ingredients | 1201061.Sweets |
| Energy drink | 1201007.Soda and mineral water |
| Treats | 1104018.Candies |
| Isotonic drink | 1201007.Soda and mineral water |
| Fruit nectar - carton, can, or bottle | 1201007.Soda and mineral water |
| Frozen Brazilian cheese puffs or ready mix | 1112025.Brazilian cheese puffs |
| Ice pop or ice cream | 1201088.Ice cream |
| Packaged sweet popcorn | 1201003.Snack |
| Ultra-processed popcorn | 1201003.Snack |
| Pizza with an ultra-processed filling | 1201003.Snack |
| Juice powder | 1114090.Juice powder |
| Regular soda | 1201007.Soda and mineral water |
| Zero sugar, low-calorie, diet soda | 1201007.Soda and mineral water |
| Fruit salad with toppings/soda | 1106.Fruits |
| Packaged salty snack, chips, savory cookie/cracker | 1112003.Cookie |
| Baked salty snack with an ultra-processed filling | 1201003.Snack |
| Fried salty snack with an ultra-processed filling | 1201003.Snack |
| Sandwich with an ultra-processed filling | 1201003.Snack |
| Tapioca with an ultra-processed filling | 1201003.Snack |

Note: UMPCP: unprocessed, minimally processed, or processed foods and culinary preparations based on these foods; UpCP: ultra-processed foods and culinary preparations based on these foods.

**Table S3** – Identification and approval by the respective ethics committees. . Food sale in Brazilian Schools (Caeb). 2022-2024.

| **Cities (Federative Units)** | **Research Ethics Committee** | **Certificate of Presentation of Ethical Appreciation** | **Report number** |
| --- | --- | --- | --- |
| Aracaju/SE | Universidade Federal de Sergipe | 58002522.3.0000.5546 | 5.531.874 |
| Belém/PA | Universidade Federal do Pará | 61830322.3.0000.0018 | 5.913.835 |
| Belo Horizonte/MG | Universidade Federal de Minas Gerais | 38003220.4.0000.5149 | 5.240.459 |
| Brasília/DF | Universidade de Brasília | 57827222.7.0000.0030 | 5.427.066 |
| Boa Vista/RR | Universidade Federal do Tocantins | 70452923.0.1001.9187 | 6.942.713 |
| Campo Grande/MS | Universidade Federal de Goiás | 71101423.4.0000.5083 | 6.796.515 |
| Cuiabá/MT | Universidade Federal do Mato Grosso | 60895722.2.0000.8124 | 5.709.474 |
| Curitiba/PR | Universidade Federal do Rio Grande do Sul | 40784920.7.0000.5347 | 6.179.413 |
| Florianópolis/SC | Universidade Federal de Santa Catarina | 71142123.6.0000.0121 | 6.172.595 |
| Fortaleza/CE | Universidade Federal de Pernambuco | 56903722.0.0000.5208 | 6.002.739 |
| Goiânia/GO | Universidade Federal de Goiás | 71101423.4.0000.5083 | 6.270.770 |
| João Pessoa/PB | Universidade Federal de Pernambuco | 56903722.0.0000.5208 | 6.002.739 |
| Macapá/AP | Universidade Federal do Tocantins | 70452923.0.1001.9187 | 6.942.713 |
| Maceió/AL | Universidade Federal de Pernambuco | 56903722.0.0000.5208 | 6.002.739 |
| Manaus/AM | Universidade Federal do Tocantins | 70452923.0.1001.9187 | 6.942.713 |
| Natal/RN | Universidade Federal de Pernambuco | 56903722.0.0000.5208 | 6.002.739 |
| Palmas/TO | Universidade Federal do Tocantins | 70452923.0.1001.9187 | 6.942.713 |
| Porto Alegre/RS | Universidade Federal do Rio Grande do Sul | 40784920.7.0000.5347 | 4.506.207 |
| Porto Velho/RO | Universidade Federal do Tocantins | 70452923.0.1001.9187 | 6.942.713 |
| Recife/PE | Universidade Federal de Pernambuco | 56903722.0.0000.5208 | 5.446.216 |
| Rio Branco/AC | Universidade Federal do Tocantins | 70452923.0.1001.9187 | 6.942.713 |
| Rio de Janeiro/RJ | UFRJ - Hospital Universitário Clementino Fraga Filho da Universidade Federal do Rio de Janeiro / HUCFF- UFRJ | 44440820.4.0000.5257 | 5.060.837 |
| Salvador/BA | Universidade Federal da Bahia | 59777922.5.0000.5023 | 5.526.372 |
| São Luís/MA | Universidade Federal de Pernambuco | 56903722.0.0000.5208 | 6.002.739 |
| São Paulo/SP | Universidade de São Paulo - Hospital das Clínicas da Faculdade de Medicina - HCFM/USP | 67365822.5.0000.0068 | 5.959.036 |
| Teresina/PI | Universidade Federal de Pernambuco | 56903722.0.0000.5208 | 6.002.739 |
| Vitória/ES | Universidade Federal do Espírito Santo | 69976923.9.1001.5060 | 6.188.500 |
